# Immune regulation based on sex differences in ischemic stroke pathology

**DOI:** 10.3389/fimmu.2023.1087815

**Published:** 2023-01-30

**Authors:** Pingping Niu, Liqin Li, Yonggang Zhang, Zhongzhou Su, Binghao Wang, He Liu, Shehong Zhang, Sheng Qiu, Yuntao Li

**Affiliations:** ^1^ Department of Neurosurgery, The Affiliated Huzhou Hospital, Zhejiang University School of Medicine (Huzhou Central Hospital), Huzhou, China; ^2^ Huzhou Key Laboratory of Basic Research and Clinical Translation for Neuro Modulation, Huzhou, China

**Keywords:** immune regulation, ischemic stroke pathology, immune cell, cytokines, sex differences

## Abstract

Ischemic stroke is one of the world’s leading causes of death and disability. It has been established that gender differences in stroke outcomes prevail, and the immune response after stroke is an important factor affecting patient outcomes. However, gender disparities lead to different immune metabolic tendencies closely related to immune regulation after stroke. The present review provides a comprehensive overview of the role and mechanism of immune regulation based on sex differences in ischemic stroke pathology.

## 1 Introduction

Stroke is well-established as the second most common cause of death worldwide (1), with significantly different outcomes between males and females (2). In this respect, current evidence suggests that females have a worse prognosis after a stroke than males, despite males having a larger incidence of stroke overall (3, 4). Generally, females are more prone to death from stroke than males (5).

An inflammatory response in the central nervous system is known as neurological inflammation. Several CNS cells, such as glial cells, endothelial cells and even immune cells in the periphery, release mediators such as cytokines, chemokines, reactive oxygen species, and secondary messengers, which play important roles in regulating CNS function. However, the immune response, a combination of innate and adaptive immune responses, is involved in normal brain growth and certain pathological conditions, including dementia and stroke (6). Inflammation caused by stroke contributes to the poor prognosis of ischemic stroke by causing neurological injury (7).

The heterogeneity in immune responses to invading and autoantigens is linked to gender differences in autoimmune illnesses, infectious disease susceptibility, vaccine efficacy and age-associated diseases, including Alzheimer’s (8, 9). It has been reported that the immune response to both foreign antigens and autoantigens is stronger in females. Current evidence suggests that females are more prone to autoimmune diseases than males (10). Compared to males, females have a higher lifetime risk of stroke, mostly during the older postmenopausal period (11). Overall, the immune systems of men and females may be fundamentally different, which may explain this discrepancy.

## 2 Gender differences in immunity

**Figure 1:**
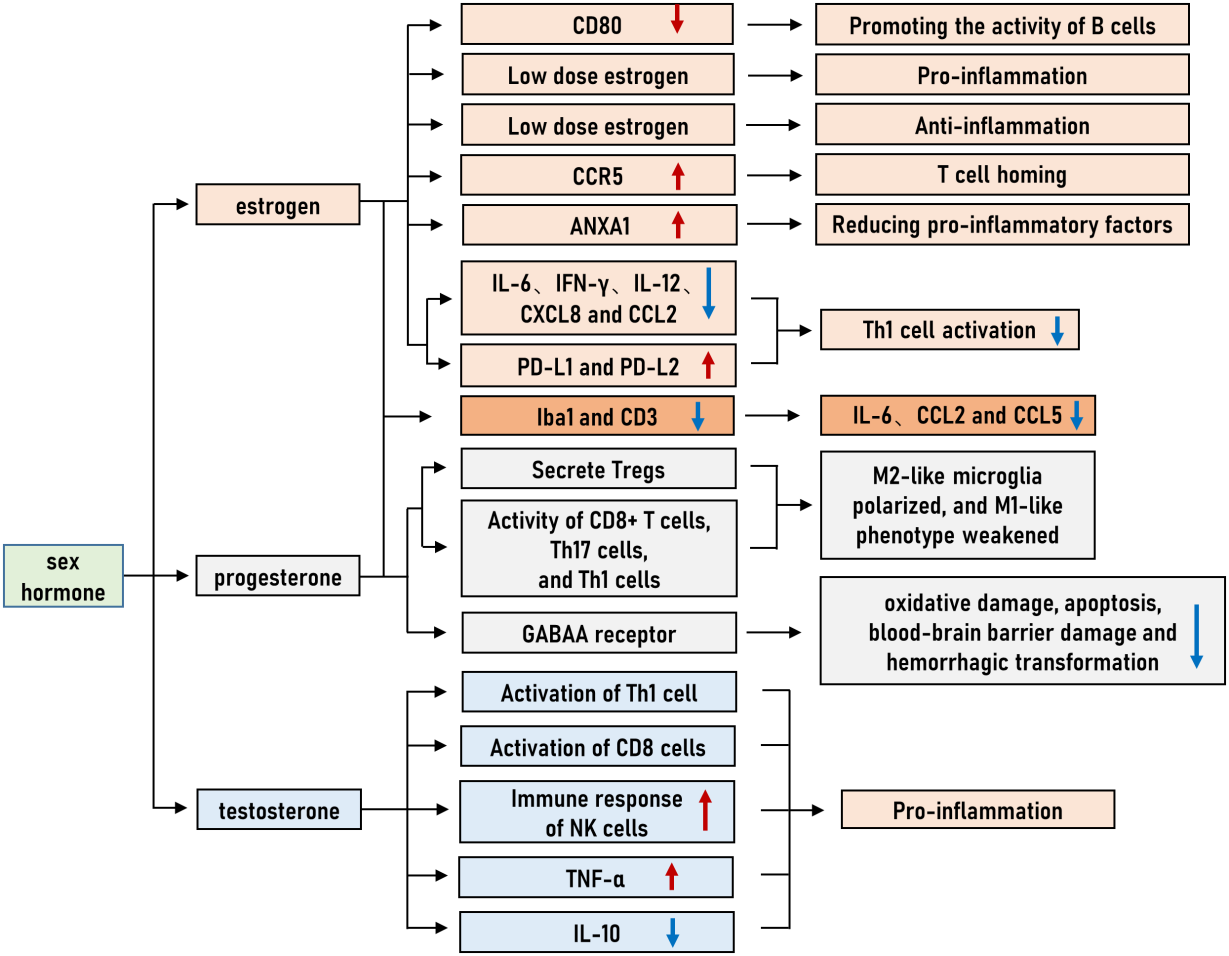
Sex difference in immunity. Estrogen, progesterone and testosterone regulate immune tendency by regulating immune cell function and cytokine release.

The immune reaction in males and females is affected by the biological differences between the sexes, which determine how immune cells react to their respective environments, as shown in **Figure 1**. Due to their higher sensitivity to antigens, robust immune response, ability to successfully fight off infections, propensity to produce inflammation, and higher prevalence of autoimmune diseases, females are more susceptible than males to autoimmune disorders (12). An increasing body of evidence suggests that females have more toll-like receptors and a higher abundance and function of monocytes, macrophages, and dendritic cells than males (13). The adaptive immune system and the Th1 response are also more active in females than in males (14). Furthermore, sex hormones influence the maturation and maintenance of the immune system. Gender disparities in immunological response have been associated with the three major gonadal hormones-estrogen, progesterone, and androgen. Estrogen receptors are expressed in T lymphocytes, B lymphocytes, natural killer (NK) cells, macrophages, and neutrophils (11, 15).

### 2.1 Sex hormone

Current evidence indicates that estrogen plays a role in neutrophil apoptosis, chemotaxis and the development of neutrophil extracellular traps (NETs) (16). Female neutrophils suppress cellular apoptosis more effectively than their male counterparts (17). The combination of estrogen and progesterone inhibits cytochrome C-mediated spontaneous apoptosis (17). Estrogen can also increase CCR5 expression, stimulating T cell homing (18). Estrogen has been shown to decrease oxidative metabolism (19), increase the production of anti-inflammatory annexin A1, block neutrophil activation (20), and decrease the release of proinflammatory cytokines such as tumor necrosis factor-α (TNF-α), Interleukin-1β (IL-1β) and IL-6 secreted by neutrophils and macrophages (21–24), which finally suppresses nuclear factor kappa-B (NF-κB) activity (25). Estrogen can decrease the expression of pro-inflammatory cytokines and chemokines, such as IL-6, IFN-γ, IL-12, CXCL8 and CCL2, up-regulate the expression of inhibitory molecules PD-L1 and PD-L2, and regulate the expression of cytokines IL-10 and TGF-β, resulting in T helper 1 (Th1) cell activation fluctuates and bias the response towards a T helper 2(Th2) phenotype (26–28). The antiviral immune response relies on plasmacytoid dendritic cells (pDC), and estrogen plays an important role in maintaining pDC homeostasis (29).

High doses of estrogen reportedly yield an immunoregulatory effect, boosting the humoral immune response to control the inflammatory response and promoting Th2 polarization and IL-4 secretion (30–32). In other words, a small amount of estrogen can cause naive T cells to develop into effector Th1 cells and produce the proinflammatory cytokine IFN-γ (33). Antibody production is correlated with estrogen dosage (34). Estrogen can promote the activity of B cells by downregulating the expression of CD80 and increasing the total amount of IgG antibodies in B cells to improve the survival rate of B cells (35).

The activation of NK cells, macrophages and dendritic cells in mice is inhibited by progesterone. Progesterone has been shown to limit the synthesis of chemokines such as macrophage inflammatory protein-1α (MIP-1α), macrophage inflammatory protein-1β, (MIP-1β) and RANTES by CD8^+^ T cells in addition to reducing the production of cytokines (36). Many immune cells, such as T cells and NK cells, have progesterone receptors on their surface (37). Progesterone, like estrogen, yields an anti-inflammatory effect by modulating the activity of microglia and astrocytes and raising the secretion of Tregs while lowering the activity of CD8^+^ T cells, T helper cells 17(Th17) and Th1 cells (38–40). In animal stroke models, progesterone has been shown to decrease infarct volume, improve neurological damage and lengthen survival time (41, 42). Reducing oxidative damage, apoptosis, blood-brain barrier breakdown, and hemorrhagic transformation (43)are some benefits of progesterone, which also controls gamma-aminobutyric acid type A (GABAA) receptors and antagonizes excitatory toxicity (42, 44).

Combination therapy with estrogen and progesterone in a middle cerebral artery occlusion (MCAO) model has been shown to protect female rats against brain injury by decreasing the expression of cortical Iba1 and CD3, as well as by inhibiting the expression of IL-6 and chemokines (CCL2, CCL5) (45). It is widely acknowledged that the peptide hormone oxytocin promotes breastfeeding and delivery. In addition to the mammary gland and the uterus, oxytocin receptors are widely distributed throughout the brain and immune system (46). Females often have higher oxytocin secretion rates than males (47). Ischemic stroke patients may benefit from oxytocin’s neuroprotective properties (48), most likely due to the hormone’s mediation of neuroimmune and anti-inflammatory properties (46).

Testosterone and dihydrotestosterone, two types of androgens, modulate the immune system by favoring the development of a Th1 response and activating CD8^+^ T cells while enhancing the NK cell response, elevating TNF-α, and decreasing IL-10 (49–51). When testosterone is present *in vitro*, the ratio of Th1:Th2 cells in male peripheral blood shifts toward more Th1 cells (52). Testosterone’s ability to bind to androgen receptors in the brain allows it to direct genetic transcription or regulate intracellular signaling pathways, affecting apoptosis, blood-brain barrier integrity, cerebral blood flow, and neuroinflammation (53, 54). Estrogens may mediate these protective effects of androgens because many androgens are aromatized into estrogens (55). When it comes to the immunological response, androgens play a key role in controlling both the innate and adaptive arms of the immune system. It is well established that androgens have immunosuppressive effects, acting on numerous arms of the immune system to dampen their activity. Androgens inhibit the immune system by acting on several immune system components (56). Androgens have also been reported to yield immunomodulatory effects. For instance, testicular-ablated males demonstrated higher surface expression of toll-like receptor 4 (TLR4) and greater vulnerability to lipopolysaccharide (LPS)-induced shock (57).

### 2.2 Chromosome

X chromosomal variations account for most of the variance in innate immune response across sexes (58). Both innate and adaptive immunological genes (such as TLR7, IRAK1, IL2RG, FOXP3, and CD40L) (58) are located on the X chromosome. The second X chromosome’s genes are normally muted; however, as people age, some of these genes can avoid being inactivated (XCI). On the X chromosome, 15% of the genes can avoid XCI (59), and their expression is higher in females (60, 61), leading to varying immunological responses (62). TLR7, TLR8 (63, 64) and the transcription factor FoxP3 in regulatory t helper cells have been linked to the innate immune response against viral infection, and females tend to have higher expression levels of these receptors than males (65). Moreover, several microRNAs on the X chromosome affect the immune system (66).

The promoters of several innate immune genes contain hormone-responsive regions (30, 67), including TLR7, MyD88, IRF7, and TLR3. Protein interactions between hormone receptors and other transcription factors that bind to DNA, such as NF-κB, specific protein 1 (Sp1), CCAAT/enhancer binding protein β(C/EBPβ) and activator protein 1 (AP-1) (31, 68), regulate gene expression and play a role in the production of proinflammatory molecules by innate immune cells. The estrogen receptor’s association with NF-κB and C/EBP β has been reported to reduce IL-6 production, indicating that sex hormones like testosterone and estrogen are crucial for innate immune responses. The Sry gene on the Y chromosome drives the growth of the testis and the generation of testosterone during the embryonic stage, demonstrating that sex chromosomes are not fully independent (58). There are counteracting effects on the immune response from chromosome complement and sex hormones (69). A study found that the immune response to autoantigens was enhanced in ovariectomized XY^Sry-^ (mice lacking Sry expression on the Y chromosome) mice compared to ovariectomized XX mice, demonstrating the usefulness of the male sex chromosome complement. However, testosterone injection dampened the immunological response, suggesting a compensatory effect between the male chromosomal complement and testosterone (69).

### 2.3 Sex differences in immune cells

#### 2.3.1 Macrophage

A dichotomy has been proposed for macrophage activation: classic vs. alternative, also M1 and M2, respectively. Like helper T cells, immunological stimulation favors the M1 polarization of male macrophages and the M2 polarization of female macrophages (70, 71). he immunological response elicited in males and females varies in intensity and nature. Macrophages are reportedly polarized toward M1 under the influence of IFN-γ and LPS and toward M2 under the influence of IL-4 or IL-13 and engaged in symbiotic relationships with other cell types to increase diversity (72, 73). These cell types include fibroblasts, mesenchymal stem cells, endothelial cells, T cells, B cells and NK cells. In this respect, mice infected with coxsackievirus B3 exhibited sex-specific differences in macrophage polarization and myocarditis severity (74). Female macrophages are associated with M2 phenotypes, while M1 activation markers are more prevalent in infected male macrophages (74). Macrophage polarization appears gender-specific in viral myocarditis, with M1 macrophages potentially damaging the host and M2 macrophages protecting against infection. In addition, the M2 phenotype macrophage has been associated to the increased susceptibility of female mice to asthma compared to male mice (75, 76). It has been shown that macrophage abundance and TLR2, TLR3 and TLR4 expression are higher, and phagocytosis is increased in females compared to males (77).

#### 2.3.2 Lymphocyte

T-cell activation in the immunological response exhibits clear sexual dimorphism. The ‘classical’ Th1 and ‘surrogate’ Th2 activation states are two of the many possible activation states of helper T cells. Th1 cell activity and secretion of related cytokines, such as IFN-γ and Interleukin-2(IL-2), are more prevalent in males, while ‘surrogate’ Th2 cell activity and the secretion of IL-4 and IL-10 are more common in females (52, 78). Mice have been found to exhibit similar sex-dependent effects, with males displaying more active Th1 responses and females displaying more active Th2 cell activity (79). Another example is the anti-inflammatory cytokine IL-10, which controls the release of T cells and Th2 cells and is associated with gender. An elevated IL-10 level is associated with a poor prognosis and immunosuppression in females, whereas this relationship is less clear in males. Stroke patients who produce high levels of IL-10 may be more susceptible to infection (80, 81). Lower levels of IL-10 production by cytotoxic T cells after stroke have been documented in males compared to females (82).

Reduced abundance of B cells throughout the body due to age and gender is associated with a loss of neurotrophic signals produced by B cells, an increase in the deleterious effects of B cell antibody production, and a decline in mental acuity (83). Besides, the protein B cell maturation antigen (BCMA) controls the division of B cells and the development of plasma cells (84). Moreover, demyelination, infiltration by inflammatory T cells and macrophages, and the severity of neuroinflammation are reportedly worse in males who lack BCMA than in females (85). Conclusions drawn from these studies suggest that the immunogenicity of the neuroinflammatory milieu is strongly influenced by gender in terms of the mechanisms governing the proliferation, survival, and differentiation of B cells. Although the overall abundance of B cells increased dramatically in women after stroke, the number of regulatory B cells decreased in the spleen compared to males (86). This discrepancy may be attributed to differences in migration to the brain. After experimental autoimmune encephalomyelitis, estrogen was found to increase the abundance of regulatory B cells in the female brain (87).

## 3 Immune regulation after stroke

The pathogenesis of the ischemic brain is mediated by the immune response following a stroke. The neuronal cell death cascade begins with the release of inflammatory signals from immune cells triggered by brain damage. Glial cells and infiltrating leukocytes, including neutrophils, monocytes, and lymphocytes, make up the bulk of the immune system. The regulation of neuronal damage and wound healing after an ischemic stroke depends on glial cell activation and the production of proinflammatory and anti-inflammatory signals. White blood cells that have entered the damage site release inflammatory mediators, which worsen brain injury.

### 3.1 Immune cell

#### 3.1.1 Microglia/macrophage

Microglia are macrophage-like cells in the CNS (88), making up around 5% - 12% of all brain cells (89). When the brain is injured, such as during a stroke, the first line of defense is microglia and activated macrophages, which release cytokines to entice even more immune cells to the injury site (90, 91). Microglia, when activated, can either promote inflammation by becoming M1-like or suppress it by becoming M2. Following an ischemic stroke, M1-like microglia release proinflammatory cytokines such as IL-1β, IL-6, and TNF-α, as well as nitric oxide synthase (92, 93), activating nuclear NF-κB and causing subsequent brain injury (94–96). Inhibiting immunity, releasing anti-inflammatory substances like IL-4 and IL-10, clearing away cell debris and misfolded proteins, encouraging extracellular matrix and tissue repair, and releasing neurotrophic factors account for the efficacy of M2-like substances in reducing inflammation (88, 92). These findings suggest that M1 and M2 microglia phenotypes contribute to the inflammatory response after a stroke (97).

Depending on the activation signals they receive, microglia can either promote injury or repair (98, 99). Different M2 subsets, such as M2a, M2b, M2c, and M2d, each have respective physiological features and biological roles (100). Strong anti-inflammatory and weak phagocytic capabilities, the ability to attract Th2 cells and drive tissue repair, etc., are all hallmarks of M2a induced by IL-4/IL-13 and mannose-CD206 receptors (100, 101). Those with an M2b phenotype exhibit increased expression of CD206, TGF-β, and CD163 and are involved in the immunological memory response and can both trigger inflammation and quell it. The M2c phenotype, associated with tissue remodeling, is triggered by glucocorticoids, IL-10 or apoptotic cells (100–102).

Although both M1 proinflammatory and M2 anti-inflammatory microglia undergo physical changes, their molecular signaling routes and activities evolve differently (93). Current evidence suggests that the early stages of ischemic stroke are characterized by predominant M2 polarization of microglia. The M1 type becomes more prevalent after a few days, particularly in the peri-infarct area (93), ultimately leading to blood-brain barrier breakdown and infiltration of peripheral immune cells (103). These multifaceted impacts of microglia/macrophages raise doubts about the efficacy of simply suppressing microglia/macrophages as a stroke treatment. Instead, rebalancing the ratio of good to negative responses by microglia/macrophages may be more effective.

#### 3.1.2 Dendritic cells and B cells

Dendritic cells are professional antigen-presenting cells that express major histocompatibility complex(MHC) II and are essential for bridging the gap between the innate and adaptive arms of the immune system (104). By presenting antigens, dendritic cells can stimulate T cell-mediated immunological responses. Resting dendritic cells have been documented close to the blood-brain barrier (105). Dendritic cells influence the migration and maturation of neighboring dendritic cells as time passes after a stroke. Most of these cells enter the ischemic area through the bone marrow (106).

Antigen presentation and antibody synthesis are functions of B cells, which are effector cells. The cerebrospinal fluid of human stroke survivors contains immunoglobulin (107, 108). Reducing the abundance of B cells in the body causes an increase in infarct size, which is associated with a poor prognosis and a high overall death rate (109). It has been found that while the early infiltration of B lymphocytes after a stroke may have a short-term favorable effect by increasing immunosuppression and the synthesis of neurotrophic factors, the long-term effects are detrimental due to an increase in autoantibodies (110). Importantly, after an ischemic stroke, IL-10-producing regulatory B cells play a protective effect (111).

#### 3.1.3 Monocytes

As incompletely differentiated cells, monocytes have potent phagocytosis abilities and the ability to mount an appropriate immune response based on their surroundings (112, 113). They can be classified into proinflammatory and anti-inflammatory fractions based on the expression of surface-specific markers. Chemotactic protein-1 (MCP-1, CCL2) and its receptor CCR2 are involved in the inflammatory response (114), and proinflammatory monocytes constitute the primary monocyte subgroups following brain injury (115),CCR2 is expressed high on proinflammatory monocytes while CX3CR1 is expressed low or not at all. As monocytes penetrate the damaged brain, they must express CCR2 in order to differentiate into macrophages. In stable environments, anti-inflammatory monocytes check blood arteries and engage in *in situ* phagocytosis (116), but they do not express CCR2. There are two main subpopulations of monocytes in rodents, distinguished by their expression of chemokine receptors and Ly-6C (Gr1). The inflammatory response is facilitated by the short half-life and active absorption of Ly6C high proinflammatory factor by the inflammatory tissue. It has been found that Ly6Clow has a long anti-inflammatory half-life and aids in vascular homeostasis (117, 118).

Blood-derived mononuclear cells undergo phenotypic and functional changes in response to the varying inflammatory conditions present during an acute ischemic stroke. The function of monocytes shifts from being proinflammatory M1 type cells to becoming anti-inflammatory M2 type cells on day 3 post-stroke (119). Through the activation of inflammasomes, M1 monocytes release reactive oxygen species (ROS), cytokines, and chemokines, and disrupt the tight connections between endothelial cells that protects the brain’s blood-brain barrier. Receptor P2X4 (P2X4R) activation enhances M1 proinflammatory phenotype polarization (120). When blood-derived mononuclear cells and other innate immune receptors collaborate, secondary inflammatory damage to the blood-brain barrier is exacerbated (121), since blood-derived mononuclear cells upregulate the triggering receptor expressed in bone marrow cells. Importantly, M2 macrophages are generated from monocytes and may shield the blood-brain barrier potentially preventing ischemia injury to the brain by vascular remodeling, physical attachment, and reduced inflammation (122, 123).

#### 3.1.4 Neutrophils

There is a favorable relationship between infarct size, stroke severity, and long-term prognosis after an ischemic stroke (124, 125). Neutrophils are white blood cells that rush to an area of illness or damage, where they consume dead cells and release inflammatory signals to draw in additional leukocytes (126). Ischemia-induced neutrophils release ROS, proteases (MMPs, protease 3, elastase), lipocalin-2(LCN-2), and NETs, all of which contribute to the breakdown of the blood-brain barrier. Connexins (primarily cadherin/β-catenin complex, occludin, ZO-1, and claudin-5) are degraded by high permeability-associated signaling pathways (e.g. MLCK, PKC, MAPK, and Rho GTPass) when there is an excess of ROS such as superoxide anion, peroxynitrite, and hydrogen peroxide (127). When it comes to the immunological response to ischemic brain injury, neutrophil adherence is a crucial first step (128). Immune cells are transported to the site of ischemic brain injury *via* the vascular wall, where they attach to adhesion molecules such as ICAM-1, MAC-1 (CD11b/CD18) and selectin (128). Within a few hours after a stroke, ICAM-1 expression rises in the proximal endothelial tissue of the brain damage, peaking at around 12–48 hours (129). In experimental stroke, infarct size and brain leukocyte infiltration have been found to be decreased in adhesion molecule deficient animals when ICAM-1 was blocked (130, 131). CD11b/CD18, also known as MAC-1, is expressed on the plasma membrane of neutrophils and binds to the intercellular adhesion molecule 1 (ICAM-1) on endothelial cells. Reduced infarct size, survival, and neutrophil infiltration into the ischemic brain are all observed in MAC-1-deficient transgenic mice after an ischemic stroke (132). P-selectin and E-selectin contribute to initial neutrophil recruitment (133), L-selectin induces recruitment of unstimulated neutrophils to activated endothelial regions (134), and selectin itself is a calcium-dependent transmembrane glycoprotein that is responsible for transporting neutrophils after cerebral ischemia (133). P- and E-selectin upregulation is positively linked with post-ischemic inflammatory response enhancement and injury severity in all experimental stroke models (135, 136).

#### 3.1.5 Lymphocyte

T-lymphocyte activation plays a role in both innate and adaptive immunity, with the ability to promote or suppress inflammation (137). It has been established that 30% of T-lymphocytes are cytotoxic T cells (CD8^+^T cells), which destroy infected cells by cytotoxic processes, while 40% are helper T cells (CD4^+^T cells), which release cytokines to modulate adaptive and innate immune responses (138, 139). Acute cerebral ischemia causes neuroinflammation, activated and infiltrated microglia/macrophages may stimulate activated CD4^+^T cells to develop into Th1 or Th2 cells, generate proinflammatory or anti-inflammatory cytokines, and either harm or protect the brain (138). Proinflammatory cytokines such as IL-2, IL-12, and IFN-γ are released by Th1 cells, which may exacerbate brain injury. Anti-inflammatory cytokines such as IL-4, Interleukin-5(IL-5), IL-10, and IL-13 may be secreted by Th2 cells, which may have a neuroprotective effect on the wounded brain (140). The earliest T cells found after a stroke are CD8^+^T cells, which detected within hours of a stroke (141). Neuronal death and exacerbated brain injury are caused by CD8^+^ T lymphocytes after they come into contact with other cells and become antigen-dependently activated, releasing perforin/granzyme (142).

To mount an immune response to an inflammatory setting, certain naive T cells can independently generate ROS and inflammatory cytokines (143). Naive CD4^+^ T cells differentiate into specific T helper cells (Th), namely Th1, Th2, Th17 and induced T regulatory cells (iTregs) (144). Maintaining immunological homeostasis and suppressing effector T cells are the functions of naturally induced Tregs (nTregs) found in the thymus and the iTregs (145). By recognizing autoantigens and foreign antigens, iTregs can suppress an overactive immune response. The post-stroke neuroinflammatory response is mitigated by Foxp3^+^Tregs (146). Toll-like receptors (TLRs) and T cell receptors (TCRs) are two examples of immunological receptors that T cells express, accounting for their immune features (147). The proinflammatory cytokine IL-17, secreted by T cells, works in tandem with IL-23 to entice monocytes and neutrophils to the site of inflammation (148).

### 3.2 Cytokines

#### 3.2.1 TNF-α

As one of the early cytokines in the inflammatory response to ischemic brain injury (149), TNF-α, is released and generated by monocytes, T cells, mast cells, macrophages, neutrophils, keratinocytes, and fibroblasts (88). Pericerebral cells are stem cells that line the surface of capillaries that TNF-α stimulates to enhance IL-6 production *via* activation of NF-κB (150, 151). Transmembrane (tmTNF-α) regulates local inflammation by cell-to-cell interaction, and soluble bioactive (sTNF-α) is created by tumor necrosis factor-converting enzyme (TACE) (88). sTNF-α acts systemically and locally in the central nervous system, promoting phagocytosis and cytotoxic activity of macrophages and enhancing the production of IL-6 and IL-1, mediated by binding of TNF-α to the receptors TNFR-1 and TNFR-2. Although TNFR1 mediates sTNF-α, TNFR-2 and TNFR-1 mediate tmTNF-α (149). The neurotoxic and neuroprotective actions of TNF-α in the ischemic brain highlight the central role of TNF-α in the neuroimmune genesis of stroke (152–154).

#### 3.2.2 IL-6

Microglia, astrocytes, leukocytes, and endothelial cells reportedly contribute to the brain injury response by releasing IL-6. There is an increasing consensus that this multi-functional proinflammatory cytokine increases leukocyte migration, regulates the production of chemokines and the expression of adhesion molecules, and activates acute phase proteins (150, 151, 155). IL-6 released after a stroke can worsen cerebral vascular damage by activating NMDI-Rs and upregulating ET-1 and JNK (156). IL-6 enhances local inflammatory responses by activating and recruiting neutrophils and monocytes and stimulating vascular endothelial cells to produce adhesion molecules and other inflammatory mediators (157). Although IL-6 is a proinflammatory cytokine, it plays a crucial role in cerebral ischemia by acting as a carrier of the inflammatory process during the early phase of stroke and as a neurotrophic factor during the late development of cerebral ischemia (158). Similar neurotrophic factors, including leukemia inhibitory factor (LIF) and ciliary neurotrophic factors, share a common receptor component, gp130 (159). Importantly, the severity of cerebral ischemia injury following an ischemic stroke may be mitigated by administering these cytokines directly into brain tissue following a stroke. Neurogenesis, angiogenesis, and neuronal differentiation are all aided by IL-6 produced by astrocytes, which also promotes Th1 polarization to Th2 and causes an immunosuppressive microenvironment (160). Additionally, IL-6 aids CNS post-traumatic recovery *via* endothelial cell repair, which may improve vascular reconstruction or angiogenesis following ischemic stroke (161). IL-6 protects neurons from apoptosis, enhances CNS neuron survival, and decreases N-methyl D-aspartate (NMDA) -mediated excitatory toxic neuron injury (158).

#### 3.2.3 IL-1

The proinflammatory cytokine IL-1 is synthesized by monocytes, macrophages, and epithelial cells (149), and the IL-1 family comprises IL-1α, IL- 1β, and IL-1RN (162). During the early stages of stroke, IL-1 mediates harmful inflammatory processes, such as the upregulation of IL-6, TNF-α, Matrix metallopeptidase 9 (MMP-9), and chemokines in astrocytes, inhibition of neurogenesis (163). IL-1 may also act on the vascular endothelium to encourage the recruitment of white blood cells (164). However, during the subacute and chronic phases of stroke, IL-1 may bring some benefits. It is widely thought that IL-1 may aid recovery from an ischemic stroke since it encourages scar formation from glial cells and boosts angiogenesis (165).

IL-1β is a cytokine that helps keep the immune system in check, and it can increase inflammation influencing nearly all the cytotypes (88). IL-1β is widely acknowledged to promote microglial activation. Indeed, microglia are pivotal in the neuroinflammatory response as effector cells. By producing potentially neurotoxic molecules like TNF-α and iNOS, they exacerbate the inflammatory response and cause secondary brain injury. The IL-1β-mediated phosphorylation and ubiquitination of inhibitor of NF-κB-a (IκB-a) by IRAK activates IκB kinase *via* the IRAK pathway, which promotes nuclear NF-κB expression and the transcription of target genes, including IL-8 and TNF-α (166). To further exacerbate injury caused by ischemia, IL-1β modulates the PI3K/AKT pathway, promotes IL-6 and other cytokines, and operates synergistically in the ischemic region (167). Growing evidence suggests that phosphorylation of JAK2/STAT3 is stimulated when IL-6 and other proinflammatory cytokines are upregulated (168, 169). Once within the nucleus, phosphorylated STAT3 (P-STAT3) upregulates the IL-1β, IL-6, and TNF-α genes by binding to certain DNA sequence features in the promoter region of the target gene (169). When brain cells are injured, it can be extremely challenging to repair the damage caused by the vicious cycle of inflammation that results.

#### 3.2.4 IL-10

IL-10 is an anti-inflammatory protein released mostly by monocytes but can also be secreted by other cell types, including Th2 lymphocytes (170). By reducing the body’s inflammatory response, IL-10 reduces the risk of stroke. By activating PI3K and STAT3, IL-10 inhibits the synthesis and activity of Th1 cells (171), reducing the expression and activity of proinflammatory cytokines such as IFN-γ, IL-1β, and TNF-α (172). Ischemic stroke is protected against thanks to IL-10 treatment’s ability to effectively down-regulate the upregulated proinflammatory signals in acute ischemic lesions (173). A study reported that transduction of the IL-10 gene prior to cerebral artery ischemia protected rat brains from ischemic and reperfused injuries by boosting heme oxygenase expression (174). Inhibiting NF-κB has been reported as another anti-inflammatory function of IL-10 (175). In a mouse model of focal cerebral ischemia (MCAO), transgenic mice overexpressing IL-32A showed decreased ischemic neuronal cell death and increased secretion of anti-neuroinflammatory factors (IL-10) by decreasing the release of neuroinflammatory factors (IL-6, IL-1β, TNF-α), thereby reducing astrocyte activation, indicating a cross-talk between IL-32 A and these other cytokines (176). Importantly, taking myelin oligodendrocyte glycoprotein (MOG) through the nose triggers Il-10 secretion from CD4^+^ T cells, which helps reduce stroke-related disability. The neuroprotective effect of oligodendrocyte glycoprotein treatment in MCAO mice (177, 178) may be due to IL-10 released by CD4^+^ T cells. Secondary infarct growth *via* the nitric oxide route may be facilitated by an increase in IL-10, which reduces the amount of CD11b^+^ cells (177).

#### 3.2.5 IL-4

The strong anti-inflammatory properties of IL-4 play an important role in determining the prognosis of stroke. By causing naive T cells to differentiate into Th2 cells that secrete anti-inflammatory cytokines, including IL-4, IL-10, and IL13, IL-4 inhibits the activity of Th1 inflammatory effector cells (179). T-cell differentiation and non-specific B-cell transformation are two immunological responses that IL-4 controls (180). Microglia/macrophage M2 polarization is promoted by IL-4, the most notable M2 macrophage polarization promoter. IL-4 is widely thought to be crucial during the acute stage of stroke (181), with dramatically increased serum levels hours after stroke onset (182). A lack of IL-4 causes brain damage and neurological impairment 24 hours after transient MCAO (183). Long-term healing after an ischemic stroke and microglia/macrophage M2 polarization depend heavily on IL-4. After cerebral ischemia, IL-4-deficient mice showed increased populations of M1-polarized microglia/macrophages and greater infarct sizes that caused neurological damage. Importantly, IL-4 recovery could reverse these effects (184). The neuroprotective effects of IL-4 are mediated by activating IL-4/STAT6 signal transduction and suppressing proinflammatory cytokines. Consistently, more proinflammatory cytokines, such as IL-1β and TNF-α, were produced by IL-4 knockout mice (185).

#### 3.2.6 Il-17

IL-23 in the brain following ischemic stroke is produced mostly by CD^172a+^/IRF4^+^ 2 dendritic cells (CDC2s), which regulate IL-17 expression in γδ T cells (186). Antigen-stimulated dendritic cells and macrophages boost Th17 cell growth during persistent inflammation by releasing interleukin-23 (186). The IL-17 produced by Th17 cells accounts for a robust inflammatory response by inducing the expression of many inflammatory cytokines. Dendritic cells migrate to the perivascular infarct area after a stroke, and cDC2s cells stimulate γδ T cells to release IL-17, which recruits neutrophils to the ischemic side of the brain (187). The synergistic effects of Vgamma4 T cell-derived IL-17A and IL-1β/IL-23 in the infarct hemisphere exacerbate the inflammatory cascade and ischemic tissue damage (188).

## 4 Sex differences in immune regulation after stroke

**Figure 2:**
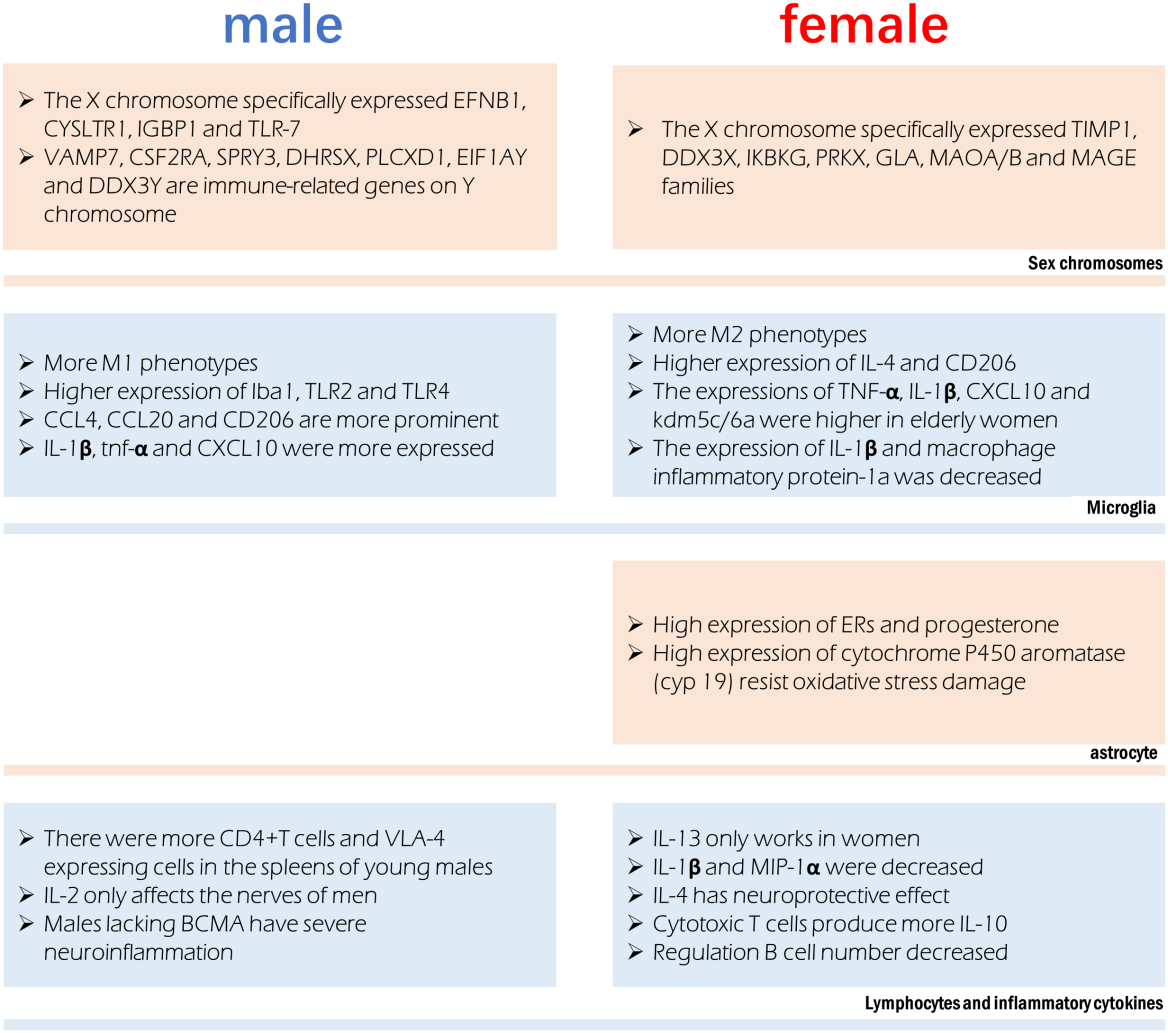
Sex difference involved in immune regulation after stroke. Different chromosomes host the expression of specific genes that can influence immune function. Microglia were more prone to the M1 phenotype in males and M2 phenotype in females, however their production of cytokines is also affected by age. Astrocytes in females have a more anti-inflammatory phenotype. Significant sex differences exist in the in lymphocytes and the cytokines they release.

Stroke treatment outcomes may vary by gender due to differences in how men and women react to inflammation throughout their lives (189, 190). The cellular and molecular mechanisms of sex hormones are distinct, summarized in **Figure 2**. Local and systemic inflammation, including the activation of glial and myeloid cells, occurs following cerebral ischemia, mostly due to the innate immune system (191, 192). Many proinflammatory genes are upregulated in response to the damage, including TNF-α, monocyte chemoattractant protein-1 (MCP-1), and IL-6 (193, 194).

### 4.1 Immune cells

In the infarct boundary zone of a stroke, microglia are the first to respond to neuronal injury as part of the innate immune response (195). The number of macrophages was lower in females and M2 phenotype microglia were higher in females, therefore showing reduced inflammation (54). Anti-inflammatory IL-4 and CD206 are more highly expressed in the ischemic brain of female microglia, and female microglia are more responsive to IL-4 and IL-10 (86, 196). Male microglia are more active and have a more proinflammatory phenotype (11, 54, 197) because they express higher amounts of Iba1, TLR2, and TLR4. Higher amounts of TNF-α, IL-1β, CXCL10, and KDM5C/6A are produced by microglia in older women, indicating a proinflammatory effect (54, 198). Experimental stroke in young male mice causes greater inflammation in the microglia (82, 199). In contrast to young male MCAO mice, female MCAO mice showed lower expression of microglial IL-1β and macrophage inflammatory protein-1a (200). Age-related variations in the hippocampus, amygdala, and cortex account for the gender gap in CCL4, CCL20 and CD206 (19). Mice of the male gender showed greater expression of genes encoding IL-1β, TNF-α, and CXCL10, among other inflammatory proteins. It has been established that genes involved in T-cell activity, adhesion molecules, cellular communication, MHC, co-stimulatory signals, cell death, and inflammatory cytokines are more highly expressed in the experimental post-stroke ischemic brains of young male mice than in females (199).

Astrocytes often play a more important role when ischemia lasts for several days. Sex hormone receptors have been documented in both male and female astrocytes. The gonadotropin hormone is responsible for the sex difference of astrocytes in ischemic stroke (201). Steroid hormones, including estradiol, progesterone, and testosterone, are largely produced by astrocytes in the brain and spinal cord (202). Both estrogen and progesterone prevent astrocyte activation (203, 204). Amphoteric astrocytes have a distinct reaction to gonadal hormones on top of the already observed differences in hormone levels between the sexes. Estrogen or an estrogen receptor (ER) agonist can trigger a positive feedback mechanism in female astrocytes, leading to increased ERs and progesterone expression (205, 206). Higher activity of estradiol-producing P450 and aromatase enzymes in female astrocytes may explain why they are more resistant to oxygen and glucose deprivation(OGD) and H_2_O_2_-induced oxidative stress than male astrocytes (207, 208). Stroke can cause neuroinflammation, and astrocytes play a role in this process, which is influenced by individual sexual hormone levels (209).

Inhibition of OGD and ROS release in astrocytes are directly induced by estrogen therapy (210), which polarizes astrocytes to an anti-inflammatory A2 phenotype (202). The expression of N-myc downstream regulatory gene 2 (Ndrg2) in astrocytes is increased by estrogen and suppresses astrocyte differentiation during ischemic stroke (211).

Peripheral leukocytes are activated after an ischemic stroke and move to the injury site, where they further cause neurological injury (13). An increasing body of evidence suggests that elimination of the spleen improves stroke prognosis in young male mouse models, where ischemia injury triggers the release of inflammatory immune cells into the periphery (13, 212, 213). Current evidence suggests an elevation in blood macrophages after a stroke, which is associated with spleen atrophy (214). The peripheral immune response to stroke is engendered at the level of the spleen and blood. Young male mice show a greater increase in CD4^+^T cells and expression of the VLA-4 adhesion molecule in the spleen than female mice do following an experimental stroke (82). A study found a significant reduction in macrophages/monocytes and activated T cells in men and a reduction in ischemic injury after splenectomy before MCAO, but these effects were not observed in women (199). Furthermore, splenectomy reduced infarct size and activated microglia in the brain in men but did not affect stroke prognosis in women (199).

### 4.2 Inflammatory cytokines

There are gender disparities in the levels of cytokines in the brain. In contrast to IL-2, which exclusively harms male nerves (215), IL-13 alleviates experimental autoimmune encephalitis symptoms in females (216), showing significant functional implications associated with gender differences in the neuroimmune system. T regulatory cells and Th2 CD4^+^ T cells produce IL-10, the inflammatory signal after stroke; however, there are gender variations in the production of these cells. The prognosis of ischemic stroke patients can be affected by post-stroke immunosuppression due to elevated IL-10 levels (80). IL-1β and MIP-1α expression were lower in microglia grown from young female MCAO mice than in male MCAO mice (217). Stroke prognosis is significantly affected by IL-4’s potent anti-inflammatory effect. Neurons produce IL-4 after an ischemic stroke and the expression of IL-4 receptors (IL-4Rs) on microglia increases in response (218). IL-4 is reportedly crucial for the neuroprotection of young female mice following stroke. Cerebral infarction is exacerbated, and the number of inflammatory cells in the brain is elevated in female mice who lack IL-4 (183). The neuroprotective benefits of IL-4 in females are mirrored by the reduced abundance of M2 microglia in the brain of IL-4 knockout mice (183).

X-chromosome genes implicated in the signaling of natural killer cells, TNFR1 signaling and axon guidance, transforming growth factor signaling, and IL17 signaling (tissue inhibitor of matrix metalloproteinase-1) have been reported to be differentially expressed in females. Genes related to development, cell trafficking, and cellular mobility experienced male-specific changes in expression, suggestive of a more potent inflammatory response in men (126). The X chromosome’s genetic manifestations in ischemic stroke exhibit significant gender heterogeneity. In this regard, it has been found that TIMP1, DDX3X, IKBKG, PRKX, GLA, MAOA/B, and MAGE gene families’ post-translational modifications, small-molecule biochemistry, and cell-cell signal transduction functions are only controlled in females (219). Only males possess the genes EFNB1, CYSLTR1, IGBP1, and TLR-7, which are involved in cell proliferation, differentiation, transport, and apoptosis (219). The XCI escape genes kdm5c and kdm6a, which demethylate H3K4me3 and H3K27me3, respectively, and epigenetically modify the expression of interferon regulatory factor (IRF4/5) (198), are responsible for changes in the proinflammatory activity of female microglia. Some genes on Y chromosomes have been reported to be differentially expressed between male stroke victims and men in general, including VAMP7, CSF2RA, SPRY3, DHRSX, PLCXD1, EIF1AY, and DDX3Y. The immune system, RNA metabolism, vesicular fusion, and angiogenesis are all regulated by processes related to the differential expression of Y chromosomal genes (220).

### 4.3 The protective effect of estrogen on the brain

Female hormones may play an important role in neuroprotection after ischemia (221, 222). In this respect, estrogen, which is generated in the brain and acts as a neuroprotective agent after stroke and an efficient anti-inflammatory agent (223), is a sex steroid hormone and a neurosteroid hormone (126). The neuroprotective effects of estrogen are mediated by its ability to dampen the immunological response that develops in the wake of brain damage caused by ischemia. Studies have demonstrated that estrogen therapy can inhibit the synthesis and secretion of proinflammatory cytokines *in vitro* and *in vivo* (25, 224). By acting as an inflammatory mediator, IL-1β is injected before estrogen is delivered to the body to precipitate the death of ischemia cells. Reduced levels of IL-1β in the rat brain’s cortex improve neurological impairment by decreasing the infarct size and blocking neutrophil infiltration into injured tissue caused by ischemia (225).

It is well-established that estrogen reduces apoptosis and oxidative stress in the brain (226), decreases NF-κB activity and the expression of IkB, iNOS, and TNF associated with ischemic neuroprotection (227–230), activates anti-apoptotic PI3K/AKT and MAPK/Erk pathways and inhibits the pro-apoptotic JNK pathway (231–233), protecting neurons from damage. Brain endothelium COX-2 induction and Il-1-mediated astrocyte activation are suppressed by estrogen (234, 235). The transcription of neurotrophic factors (IGF-1, BDNF, GDNF, and VEGF) is regulated by estrogen, reducing neuroinflammation. Importantly, estrogen also enhances the transcription of STAT3 and PPAR, inhibiting NF-κB transcriptional activity (236–240).

Low concentrations of TNF aid in promoting injury repair, while high concentrations are neurotoxic (241, 242). Estrogen regulates the production of the proinflammatory cytokine TNF, which acts on receptors of many cell types, including neurons, glial cells, and endothelial cells (243). One such inflammatory protein that estrogen regulates is nuclear NF-κB, which modulates inflammatory signaling pathways in cells, including neurons (244). After ischemia, NF-κB regulates the upregulation of inflammatory mediators, including IL-1, IL-6, IL-8, iNOS, ICAM 1, VCAM and E-selectin, and then leads to neutrophil infiltration and adhesion molecule induction. Estrogen treatment can inhibit the activation of NF-κB, reduce the activation of NF-κB-mediated delayed cell death in ischemia-reperfusion injury, alleviate inflammation and apoptosis, and protect neurons (225).

## 5 Conclusion

The role of sex differences in the incidence and prognosis of ischemic stroke is significant, and the immune regulation based on sex difference is one of the important reasons. However, the importance of gender factors varies at different ages, which is due to sex hormone levels, underlying disease differences and other factors. There is growing evidence that some risk factors, like hypertension and atrial fibrillation, tend to occur more frequently in older women, while others, like diabetes and smoking, disproportionately affect women. The disparity in stroke prevalence and outcome may also be influenced by sex-specific risk factors, such as the use of oral contraceptives and menopause. Evidence suggests that females are more likely than males to exhibit non-traditional acute stroke symptoms, making it more challenging for clinicians to correctly diagnose a stroke and potentially delaying the administration of thrombolytic intervention.

More regrettably, there is still no gender-specific stroke treatment, even though gender differences are a valuable risk factor in many studies. Research on immune regulation based on sex difference presents great difficulties because it is difficult to simulate human ischemic stroke state *in vitro* as well as in animal models, leading to a slow progress in the study of mechanisms in this area. We expect more detailed studies to support the development of gender-specific stroke treatment options, including immunotherapy agents and specific inflammatory markers.

## Author contributions

PN and LL completed the manuscript and contributed equally to the article. YZ, ZS, BW, HL, and SZ collected and collated literature. SQ and YL designed this work. All authors contributed to the article and approved the submitted version.
